# Role of sex on the efficacy of pharmacological and non-pharmacological treatment of heart failure with reduced ejection fraction: A systematic review

**DOI:** 10.3389/fcvm.2022.921378

**Published:** 2022-07-25

**Authors:** María Ascensión Sanromán Guerrero, Sonia Antoñana Ugalde, Elena Hernández Sánchez, Susana del Prado Díaz, Marta Jiménez-Blanco Bravo, David Cordero Pereda, José Luis Zamorano Gómez, Jesús Álvarez-García

**Affiliations:** Department of Cardiology, Ramón y Cajal University Hospital, IRYCIS, Centro de Investigación en Red en Enfermedades Cardiovasculares (CIBERCV), Madrid, Spain

**Keywords:** sex differences, gender, heart failure, women, sex

## Abstract

**Background:**

Heart Failure (HF) is a growing epidemic with a similar prevalence in men and women. However, women have historically been underrepresented in clinical trials, leading to uneven evidence regarding the benefit of guideline-directed medical therapy (GDMT). This review aims to outline the sex differences in the efficacy of pharmacological and non-pharmacological treatment of HF with reduced ejection fraction (HFrEF).

**Methods and results:**

We conducted a systematic review *via* Medline from inception to 31 January 2022, including all randomized clinical trials published in English including adult patients suffering HFrEF that reported data on the efficacy of each drug. Baseline clinical characteristics, primary outcomes, and sex-specific effects are summarized in tables. The systemic review has been conducted in accordance with the Preferred Reporting Items for Systematic Reviews and Meta-analyses (PRISMA) statement. In total, 29 articles were included in the systematic review. We observed that the proportion of women enrolled in clinical trials was generally low, the absence of a prespecified analysis of efficacy by sex was frequent, and the level of quality of evidence on the efficacy of GDMT and implantable cardioverter defibrillator (ICD) or cardiac resynchronization therapy (CRT-) in women was relatively poor.

**Conclusions:**

Sex influences the response to treatment of patients suffering from HFrEF. All the results from the landmark randomized clinical trials are based on study populations composed mainly of men. Further studies specifically designed considering sex differences are warranted to elucidate if GDMT and new devices are equally effective in both sexes.

## Introduction

Heart failure (HF) is a global epidemic that is growing every year, with a similar prevalence and incidence in men and women (1, 2). During the last 30 years, there has been a significant advance in the treatment of HF, in particular in those patients suffering from HF with reduced ejection fraction (HFrEF) (3). Thus, current guidelines recommend several saving-life therapies, such as drugs and devices, based on the positive results of randomized clinical trials (4, 5).

However, women have been underrepresented in every landmark study, preventing us from concluding if the benefit of these therapies is unequivocally observed in both sexes (6). There are also sex differences in demographics and pathophysiology which may modulate the response to HF treatments (7). Moreover, some social factors historically linked to gender have determined distinct patterns in clinical presentation, workup, and management in HF that, in turn, also could play a role in the treatment of women (8). In consequence, greater awareness about the relevance of closing these gaps and implementing strategies that consider a sex perspective is rising from the scientific community to medical societies (9, 10).

The purpose of this systematic review is to describe the sex-specific differences in the efficacy of pharmacological and non-pharmacological treatment of HFrEF.

## Methods

This review was reported according to the Preferred Reporting Items for Systematic Reviews and Meta-Analyses (PRISMA) statement (11).

### Search strategy

An electronic systematic review of the literature was conducted in the Medline database (National Library of Medicine Bethesda, Maryland). The keywords used were chosen according to the MESH terminology: (sex OR gender OR female OR male OR women OR men) AND (beta-blocker OR Nebivolol OR Bisoprolol OR Metoprolol OR Carvedilol OR sacubitril OR sacubitril-valsartan OR angiotensin neprylisin inhibitor receptor OR sodium glucose cotransporter inhibitor OR SGLT2 inhibitor OR dapagliflozin OR empagliflozin OR sotagliflozin OR mineralocorticoid receptor antagonist OR MRA OR eplerenone OR spironolactone OR ivabradine OR implantable cardioverter defibrillator OR ICD OR cardiac resynchronization therapy OR CRT). These terms were restricted to “Title/Abstract” and “English” (Language). The search was conducted from inception to 31 January 2022. In addition, we conducted a hand-searching of reference lists of all included studies and guidelines to identify further studies.

### Eligibility criteria for study selection and validity assessment

The inclusion criteria were the following: (i) randomized clinical trials including adult patients (≥18 years of age) suffering from HFrEF and (ii) studies that reported data on the efficacy of each drug. There was no restriction on the publication date. We excluded animal studies, abstracts, editorials, commentaries, systematic reviews, and narrative reviews. Once duplicates were removed, all authors independently screened titles and abstracts to ensure the capture of all relevant studies. Disagreements were resolved by discussion to achieve consensus.

### Data extraction and outcomes of interest

Data were extracted by the authors into predetermined tables using a standardized protocol. The data extracted were drug name, study name, year of publication, characteristics of the study population, number of included patients, number of women included, left ventricular ejection fraction (LVEF), efficacy primary endpoint, sex-specific outcomes, and *p*-value for interaction when available. The primary outcomes of interest were all-cause mortality or the combined endpoint of mortality and HF hospitalization. This systematic review was restricted to data published in manuscript or abstract form. We expressed study results as relative risk (*RR*) or hazard ratio (*HR*) with 95% confidence intervals (*CI*) when available.

## Results

### Study selection

**Figure 1:**
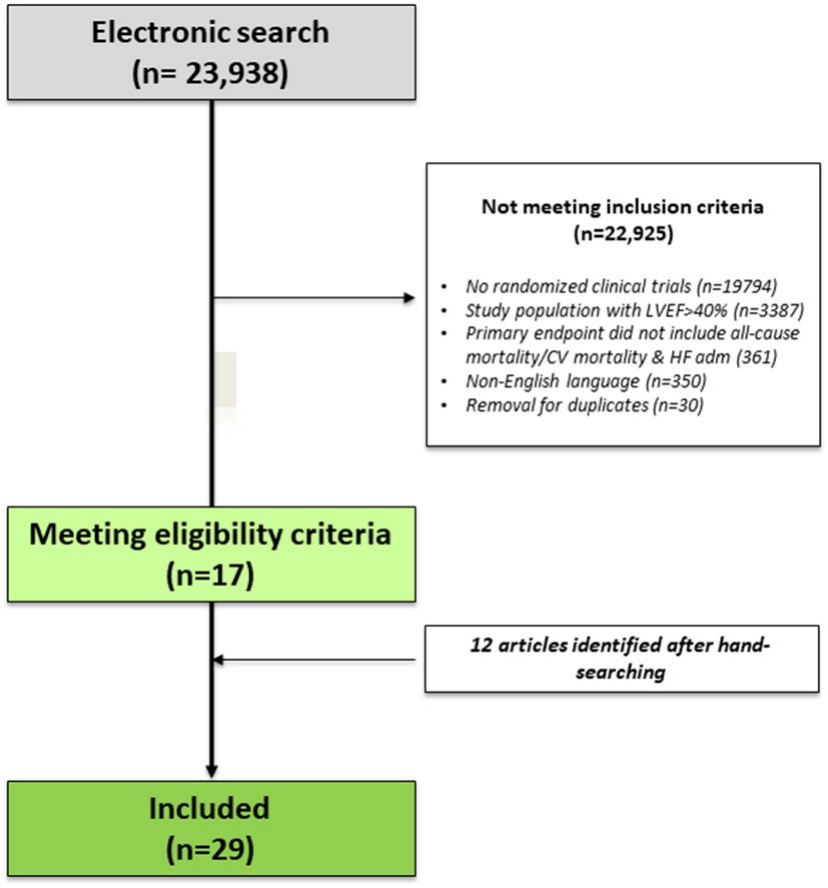
Flow-chart of the study selection.

Our electronic search retrieved 23,938 articles. After the removal of duplicates and those which did not fulfill inclusion criteria, 17 articles were identified. After hand-searching, 12 articles were identified. Finally, 29 articles were included in our systematic review. Figure 1 illustrates the flowchart of the study selection. The main characteristics of the included studies are summarized in Table 1. The specific results of the studies are presented in chronological order of appearance by drug class.

**Table 1:** Randomized clinical trials for drugs in HFrEF included in the systematic review.

**Intervention**	**Study name**	**Year**	**Study population**	***N***	**Women (%)**	**LVEF**	**Primary endpoint**	**Overall treatment effect (95% CI)**	**Sex-specific effect**	***P*** **value for sex interaction**
Enalapril	CONSENSUS	1987	NYHA IV Congestive HF	253	74 (30)	–	All-cause mortality	RR 0.56 (0.34–0.91)	Not performed	–
Enalapril	SOLVD	1991	NYHA I-IV Congestive HF (90% NYHA II-III)	2569	504 (20)	≤ 35%	All-cause mortality	RR 0.86 (0.74–0.95)	Not performed	–
Carvedilol	US Carvedilol HF	1996	NYHA I–IV	1094	256 (23)	≤ 35%	All-cause mortality	HR 0.35 (0.20–0.61)	HR 0.41 (0.22–0.80) in men; HR 0.23 (0.07-0.69) in women	Not reported
Bisoprolol	CIBIS II	1999	NYHA III–IV	2647	515 (19)	≤ 35%	All-cause mortality	HR 0.66 (0.54–0.81)	HR 0.53 (0.42–0.67) in men; HR 0.37 (0.19–0.69) in women	Not reported
Metoprolol	MERIT-HF	1999	NYHA II–IV	3991	898 (23)	≤ 40%	All-cause mortality	RR 0.66 (0.53–0.81)	HR 0.61 in men (*p* <0.001); HR 0.92 in women (*p* = NS)	0.14
Bucindolol	BEST	2001	NYHA III–IV	2708	593 (22)	≤ 35%	All-cause mortality	HR 0.90 (0.78–1.02)	No differences among sexes	Not reported
Carvedilol	COPERNICUS	2001	NYHA III–IV	2289	469 (20)	<25%	All-cause mortality	HR 0.65 (0.52–0.81)	Significant benefit in men, trend toward benefit in women	Not reported
Valsartan	Val-HeFT	2001	NYHA II–IV	5010	1003 (20)	<40%	Mortality or cardiac arrest or HF admission or need for iv therapy	RR 0.87 (0.77–0.97)	Significant benefit in men, trend toward benefit in women	Not reported
Candesartan	CHARM added	2003	NYHA II–IV + ACEI	2548	542 (21)	≤ 40%	CV death or HF admission	HR 0.85 (0.75–0.96)	No differences among sexes	0.87
Candesartan	CHARM alternative	2003	NYHA II-IV, intolerant to ACEI	2028	646 (32)	≤ 40%	CV death or HF admission	HR 0.77 (0.67–0.89)	No differences among sexes	0.87
Spironolactone	RALES	1999	NYHA III-IV	1663	446 (27)	≤ 35%	All-cause mortality	RR 0.70 (0.60–0.82)	No differences among sexes	Not reported
Eplerenone	EPHESUS	2003	Acute MI and HF or diabetes mellitus	6632	1918 (29)	≤ 40%	All-cause mortality	RR 0.85 (0.75–0.96)	Significant benefit in women, trend toward benefit in men	0.44
Eplerenone	EMPHASIS-HF	2011	NYHA II and older than 55 years old	2737	610 (22)	≤ 35%	CV death/HF admission	HR 0.63 (0.54–0.74)	No differences among sexes	0.36
Ivabradine	SHIFT	2010	NYHA II-IV	6505	1535 (24)	≤ 35%	CV death/HF admission	HR 0.82 (0.75–0.90)	No differences among sexes	0.26
Sacubitril-valsartan vs enalapril	PARADIGM	2014	NYHA II-IV	8399	1832 (22)	≤ 40%	CV death/HF admission	HR 0.80 (0.73–0.87)	No differences among sexes	0.63
Dapagliflozin	DAPA-HF	2019	NYHA II-IV	4744	1109 (23)	≤ 40%	CV death/Worsening HF	HR 0.74 (0.65–0.85)	HR 0.73 (0.63–0.85) in men, HR 0.79 (0.59–1.06) in women	0.67
Empagliflozin	EMPEROR-Reduced	2020	NYHA II–IV	3730	893 (24)	≤ 40%	CV death/Worsening HF	HR 0.75 (0.65–0.86)	HR 0.80 (0.68–0.93) in men, HR 0.59 (0.44–0.80) in women	Not reported

*HFrEF, heart failure with reduced ejection fraction; LVEF, left ventricular ejection fraction; NYHA, New York Heart Association; HF, heart failure; RR, relative risk; HR, hazard ratio; CV, cardiovascular; ACEI, angiotensin-converter enzyme inhibitor; MI, myocardial infarction*.

### Angiotensin-converting enzyme inhibitors

Two studies were reviewed according to the inclusion criteria (12, 13). The Cooperative North Scandinavian Enalapril Survival (CONSENSUS) study was conducted in 1987 to evaluate the impact of Enalapril vs. placebo in 253 patients with the New York Heart Association (NYHA) IV congestive HF. Only 74 (30%) patients were women and, interestingly, LVEF was not measured. After a 6-month follow-up period, enalapril significantly reduce all-cause mortality, but a sex-based analysis was not performed (12). After 4 years, the Studies of Left Ventricular Dysfunction (SOLVD) analyzed the effect of Enalapril vs. placebo in 2,569 patients with mostly NYHA II-III congestive HF and LVEF ≤ 35% (505 women, 20%), showing a significant 14% risk reduction of death at 4-year. However, an analysis stratified by sex was not performed either (13).

Two meta-analyses including the CONSENSUS and SOLVD study populations together with smaller studies showed that the mortality benefit of ACEI showed only a trend for benefit in women, without reaching statistical significance (14, 15).

### Beta-blockers

Five studies were finally selected according to our selection criteria (16–20). The U.S. Carvedilol of HF study was conducted in 1996 in 1,094 patients (256 women, 23%) suffering from chronic HF with LVEF ≤ 35% and showed that carvedilol significantly reduced the risk of death by 65% after a median follow-up of 6.5 months. The analysis stratified by sex showed similar benefits in both sexes (16). The CIBIS II was a trial performed in 1999 to assess bisoprolol vs. placebo on all-cause mortality in 2,564 patients (515 women, 19%) with advanced HF and LVEF <35% already treated with ACEI (17). It was also stopped early because a clear benefit was observed in the group assigned to beta-blockers. Women differed from men with regard to age, the NYHA functional classification, the primary cause of HF, and risk factors, such as left bundle-branch block. In a *post-hoc* analysis, bisoprolol reduced the mortality rates for both men and women after adjustment for baseline differences (21). The MERIT-HF was another clinical trial conducted in 1999 in 3,991 (898 women, 23%) patients with advanced HF and LVEF ≤ 40% to investigate whether metoprolol-controlled release/extended release (CR/XL) once daily added to optimum standard therapy lowered mortality (18). After a median follow-up of 1 year, a 34% decrease in death risk was observed in the metoprolol arm. In a *post-hoc* analysis, treatment with metoprolol CR/XL in women resulted in a 21% reduction in the primary combined endpoint of all-cause mortality/all-cause hospitalizations (164 vs. 137 patients; *p* = 0.044) (22). In the Beta-blocker Evaluation of Survival Trial (BEST), it was evaluated bucindolol vs. placebo in 2,708 patients (593 women, 23%) with NYHA III or IV HF and LVEF ≤ 35% (19). The primary endpoint to evaluate was death from any cause and the results showed no improvement in survival. In a prespecified analysis by sex, no differences were observed among men and women (23). The Carvedilol Prospective Randomized Cumulative Survival (COPERNICUS) trial was designed to evaluate the effects of carvedilol in 2,289 patients (465 women, 20%) with severe chronic HF and LVEF ≤ 25% (20). Carvedilol reduced the combined endpoint of death or hospitalization among the 469 women studied, mostly driven by a reduction in hospitalization, but the significant reduction in all-cause death was only achieved in men.

In a pooling-data analysis of total mortality by sex from CIBIS II, MERIT-HF, and COPERNICUS, beta-blockers showed very similar and statistically significant survival benefits in women (*RR* 0.69; 95% *CI* 0.51–0.93) and men (0.66; 95% *CI* 0.58–0.75) (22).

### Antagonist receptor blockers

Three studies were reviewed according to the inclusion criteria (24–26). The Valsartan Heart Failure Trial (Val-HeFT) was conducted in 2001 to evaluate the effect of valsartan vs. placebo on mortality in 5,010 patients (1,003 women, 20%) with NYHA II-IV HF and LVEF <40% (24). On top of ACEI, diuretics, digoxin, and beta-blocker treatment, valsartan significantly reduced the combined endpoint of mortality or cardiac arrest, HF hospitalization, or need for intravenous therapy. There was a clear benefit in men and a trend toward benefit in women, although it did not reach statistical significance. In a *post-hoc* analysis adjusted for NYHA class, LVEF, use of ACEI and beta-blockers, and HF etiology, valsartan reduced the adjusted *RR* for the combined endpoint in women (0.84; 95% *CI*: 0.67–1.06; *p* = 0.044), but not in men (0.872; 95% *CI*: 0.779–0.975; *p* = 0.053) (27). The Candesartan in Heart failure Assessment of Reduction in Mortality and Morbidity (CHARM) program was specifically designed as three double-blind, placebo-controlled, clinical trials comparing candesartan vs. placebo in three distinct populations with symptomatic HF. In those two trials including subjects with LVEF ≤ 40% (being treated with an ACEI -CHARM-Added- or intolerant to ACEI -CHARM-Alternative-), candesartan significantly reduced the combined endpoint of cardiovascular death or HF readmission (25, 26). This reduction was similar in men and women (28).

### Mineraloid receptor antagonists

Three studies met the established search criteria for drugs (29–31). The Randomized Aldactone Evaluation Study (RALES) was a trial to test the hypothesis that daily treatment with spironolactone would significantly reduce the risk of all-cause death among 1,663 patients (446 women, 27%) who had severe HF and LVEF ≤ 35% who were receiving standard therapy, such as ACEI. The RALES accomplished the primary endpoint, with a similar benefit in both sexes, and a good safety profile (29). The Eplerenone Post–Acute Myocardial Infarction Heart Failure Efficacy and Survival Study (EPHESUS) was a trial to evaluate the effect of eplerenone—an aldosterone blocker that selectively blocks the mineralocorticoid receptor—on overall mortality in 6,632 patients (1,918 women, 29%) with acute myocardial infarction complicated by left ventricular dysfunction and HF who were receiving optimal medical therapy. Eplerenone also met the primary endpoint for efficacy, but regarding sex-specific effects, women presented a higher benefit than men for mortality risk reduction (30). Lastly, the Eplerenone in Mild Patients Hospitalization and Survival Study in Heart Failure (EMPHASIS-HF) was designed to investigate the effects of eplerenone, added to evidence-based therapy, on clinical outcomes in 2,737 patients (610 women, 22%) with NYHA II HF and LVEF ≤ 35% (31). After a median follow-up of 21 months, patients allocated in the drug arm showed a significant 37% reduction in the primary endpoint composed by cardiovascular death or HF admission and a significant 24% reduction in all-cause mortality. Similar benefits were observed among men and women.

### Ivabradine

The Systolic Heart failure treatment with the If inhibitor ivabradine Trial (SHIFT) study reported a significant reduction in the composite endpoint of cardiovascular death or HF hospitalization with ivabradine vs. placebo (*HR* 0.82, 95% *CI* 0.75–0.90, *p* < 0.0001) in 6,505 patients (1,535 women, 24%) with symptomatic HF and LVEF ≤ 35%, in sinus rhythm and with heart rate ≥70 beats per minute (bpm) (32). The effects were driven mainly by hospital admissions for worsening HF (*HR* 0.74, 0.66–0.83; *p* < 0.0001) and deaths due to HF (*HR* 0.74, 0.58–0.94, *p* = 0.014). This lower rate of the composite endpoint with ivabradine was similar in both sexes (*p*-value for interaction = 0.260).

### Angiotensin receptor neprylisin inhibitor

Only the Prospective Comparison of Angiotensin receptor neprylisin inhibitor (ARNI) with ACEI to Determine Impact on Global Mortality and Morbidity in Heart Failure Trials (PARADIGM-HF) met the inclusion criteria (33). This was a clinical trial conducted in 2014 which evaluated sacubitril-valsartan (SV) vs. enalapril in 8,399 patients (1,832 women, 22%) with NYHA II–IV, LVEF ≤ 40% and increased natriuretic peptides. After a median follow-up of 27 months, patients allocated in the SV arm showed a significant 20% reduction in the primary endpoint composed by cardiovascular death or HF admission and a significant 16% reduction in all-cause mortality. Similar benefits were observed in both sexes.

### Sodium-glucose cotransporter 2 inhibitors

Two clinical trials met the search criteria (34, 35). The Dapagliflozin and Prevention of Adverse Outcomes in Heart Failure (DAPA-HF) prospectively evaluated the efficacy and safety of dapagliflozin in 4,744 patients (1,109 women, 23%) with NYHA II–IV and LVEF ≤ 40%, regardless of the presence of diabetes (34). Over a median of 18 months, the primary outcome (worsening HF or CV death) occurred in 386 of 2,373 patients (16.3%) in the dapagliflozin group and in 502 of 2,371 patients (21.2%) in the placebo group (*HR*, 0.74; 95% *CI* 0.65–0.85; *p* < 0.001). Moreover, a total of 276 patients (11.6%) in the dapagliflozin group and 329 patients (13.9%) in the placebo group died from any cause (*HR* 0.83; 95% *CI*, 0.71–0.97). In a prespecified subgroup analysis of the DAPA-HF, dapagliflozin reduced the risk of worsening HF, CV death, and all-cause death and improved symptoms, physical function, and health-related quality of life similarly in men and women with HFrEF. In addition, dapagliflozin was safe and well-tolerated irrespective of sex (36). The Empagliflozin Outcome Trial in Patients with Chronic Heart Failure and a Reduced Ejection Fraction (EMPEROR-Reduced) evaluated empagliflozin in 3,730 patients (893 women, 24%) with NYHA II–IV and LVEF ≤ 40%, regardless of the presence of diabetes (35). After a median follow-up of 16 months, the primary outcome event occurred in 361 of 1,863 patients (19.4%) in the empagliflozin group and in 462 of 1,867 patients (24.7%) in the placebo group (*HR* for CV death or hospitalization for HF, 0.75; 95% *CI* 0.65–0.86; *p* < 0.001). The effect of empagliflozin on the primary outcome was consistent in patients of both sexes.

### Implantable converter defibrillators and cardiac resynchronization therapy

In total, 12 clinical trials were reviewed according to inclusion criteria (37–48). Table 2 summarizes the main characteristics of the randomized clinical trials in HfrEF for implantable cardioverter defibrillator (ICD) and cardiac resynchronization therapy (CRT).

**Table 2:** Randomized clinical trials for ICD/CRT in HFrEF included in the systematic review.

**Intervention**	**Study name**	**Year**	**Study population**	**N**	**Women (%)**	**LVEF**	**Primary endpoint**	**Overall treatment effect (95% CI)**	**Sex-specific effect**	***P*** **value for sex interaction**
ICD	MADIT II	2002	Prior MI	1232	192 (16)	≤ 30%	All-cause mortality	HR 0.69 (0.51–0.93)	HR 0.66 (0.48–0.91) in men, HR 0.57 (0.28–1.18) in women	0.72
ICD	AMIOVIRT	2003	NIDCM and asymptomatic NSVT	103	30 (29)	≤ 35%	All-cause mortality	1- and 3-year survival rates did not differ between both arms (*p* = 0.8)	Not performed	–
ICD	DINAMIT TRIAL	2004	Post-acute MI patients	694	160 (24)	≤ 35%	All-cause mortality	HR 1.08 (0.76–1.55)	Not reported	0.82
ICD	DEFINITE TRIAL	2004	Non-ischemic dilated cardiomyopathy with PVB	458	264 (29)	≤ 35%	All-cause mortality	HR 0.65 (0.40–1.06)	HR 0.49 (0.27–0.90) in men, HR 1.14 (0.50–2.64) in women	0.18
ICD	SCD HeFT	2005	NYHA class II or III	2521	588 (23)	≤ 35%	All-cause mortality	HR 0.77 (0.62–0.96)	HR 0.73 (0.57–0.93) in men, HR 0.96 (0.56–1.61) in women	0.54
ICD	IRIS TRIAL	2009	Post-acute MI patients with HR ≥ 90 bpm	898	209 (23)	≤ 40%	All-cause mortality	HR 1.04 (0.81–1.35)	Not reported	0.85
ICD	DANISH	2016	NIDCM	1160	307 (28)	≤ 35%	All-cause mortality	HR 0.87 (0.68–1.12)	HR 0.85 (0.64–1.12) in men, HR 1.03 (0.57–1.87) in women	0.66
CRT	COMPANION	2004	NYHA III-IV and a QRS ≥ 120 ms	1520	493 (33)	≤ 35%	Time to death from or hospitalization for any cause	CRT vs OMT: HR 0.81 (*p* = 0.014) ICD-CRT vs OMT: HR 0.80 (*p* = 0.01)	Not reported	Not reported
CRT	MADIT-CRT	2005	Cardiomyopathy with QRS≥130 msec and NYHA I-II	1820	453 (25)	≤ 30%	All-cause mortality and HF events	HR 0.66 (0.52–0.84)	HR 0.76 (0.59–0.97) in men, HR 0.37 (0.22–0.60) in women	0.01
CRT	CARE HF	2005	NYHA III-IV and cardiac desynchrony	813	216 (27)	≤ 35%	Time to death from any cause or an unplanned hospitalization for a major cardiovascular event	HR 0.63 (0.51–0.77)	HR 0.62 (0.49–0.79) in men, HR 0.64 (0.42–0.97) in women	Not reported
CRT	RAFT	2010	NYHA II-III, QRS ≥120 ms or a paced QRS duration ≥ 200 ms	1798	308 (18)	≤ 30%	Death from any cause or hospitalization for HF	HR 0.75 (0.64–0.87)	Not reported	0.09
CRT	ECHO-CRT	2013	NYHA III-IV, QRS <130 ms and desynchrony	809	227 (28)	≤ 35%	Death from any cause or first hospitalization for HF	HR 1.20 (0.92–1.57)	HR 1.31 (0.95–1.80) in men, HR 0.93 (0.56–1.56) in women	0.43

*ICD, implantable cardioverter defibrillator; CRT, cardiac resynchronization therapy; HFrEF, heart failure with reduced ejection fraction; LVEF, left ventricular ejection fraction; NYHA, New York Heart Association; HF, heart failure; NIDCM, non-ischemic dilated cardiomyopathy; NSVT, non-sustained ventricular tachycardia; PVB, premature ventricular beats; RR, relative risk; HR, hazard ratio; CV, cardiovascular; MI, myocardial infarction*.

Several randomized trials have proven the efficacy of ICD to prevent all-cause death. As in most HF drug therapy trials, women were underrepresented in these studies, accounting for less than one-third of the total population (37–43). Overall, subgroup analyses of each study were consistent and did not show statistically significant differences between both sexes.

On the other hand, CRT studies included a wide variety of patients (with NYHA classes ranging from I to IV), but less than one-third of them were women. The subgroup analysis of most trials did not show a significant difference in outcomes between men and women (44–48). An exception to this is the MADIT-CRT trial, in which ICD plus CRT therapy was associated with a greater benefit in women (*p* for interaction = 0.01) (49).

## Discussion

### Main findings

In this systematic review including 28 randomized clinical trials evaluating pharmacological and non-pharmacological treatment of HFrEF, we observed that: (1) the proportion of women enrolled was generally low, (2) the absence of a prespecified analysis of efficacy by sex was frequent, and (3) the level of quality of evidence on the efficacy of GDMT and ICD or CRT in women is relatively poor.

### Role of sex on HFrEF treatment

Over the last 30 years, many significant advances have been made in the treatment of patients suffering from HFrEF. Thus, the main HF guidelines that have been recently published recommend starting neurohormonal drugs and SGLT2 inhibitors at the same level to achieve the maximum mortality risk reduction (4, 5). Once GDMT is implemented at the highest tolerated dose and LVEF is again assessed, ICD and CRT have to be considered in those patients with an estimated survival greater than 1 year according to HF etiology, morphology, and duration of QRS complex.

However, this “foundational therapy” approach is not supported by the same level of quality of evidence when sex is considered. After reviewing the principal landmark trials involving drugs, we observed that women were repeatedly underrepresented and prespecified sex-based analyses were not performed. Only in the case of the most recent families, sacubitril-valsartan and SGLT2 inhibitors, we should be confident that the sex interaction was not significant when assessing the efficacy of the drug (33–36).

This uneven supporting evidence is particularly relevant when epidemiological, physiological, and pharmacological differences by sex are known. The HF incidence increases over time with aging in both sexes and the overall lifetime risk for developing HF is also similar (50, 51). Nevertheless, women tend to be older, with a higher prevalence of comorbidities than men when HFrEF appears (52). In addition, the presence of risk factors is different according to sex (less smoking and more diabetes in women) and the social determinants of health can also be especially unique in women (7). Regarding to pathophysiological differences by sex, the predisposition to macrovascular coronary artery disease and myocardial infarction in men may only explain a part of the higher risk of HFrEF compared with women (6). As we said, HFrEF in women is more likely to be present with aging and non-cardiac comorbidities, and distinct immune responses can be particularly important when inflammation and microvascular disease are pointed out to develop HF (53, 54). Specific etiologies of HFrEF, such as Takotsubo syndrome, peripartum cardiomyopathy, or cardiotoxicity (whether related to chemotherapy or alcohol abuse) also involve different consequences by sex (7, 55). Lastly, there are relevant sex-differences in pharmacokinetics and pharmacodynamics based on differences in body composition (with women usually having lower weight and height, a higher proportion of body fat, and a lower peripheral distribution volume) and lower renal and hepatic filtration rate (56). Several studies have suggested that the maximum benefit of GDMT may be achieved in women at doses lower than those recommended by the guidelines (57).

In relation to devices, women are less likely to receive an ICD than men, but they have higher rates of device implantation-related complications. Instead, women are more likely to respond favorably to CRT than men, which can result in an improvement of survival rates. The reasons for this are not clear still but include differences in vascular access, higher hemorrhagic risk, QRS duration cutoff, and less ischemic HF origin (58).

### Limitations

Our systematic review has some limitations. First, we only included randomized clinical trials in our study. Although publication and selection bias may arise because we selected those published in English, the main pivotal studies are usually published in this language. Second, since only aggregated data were available, it was not possible to perform a more granular analysis of clinical outcomes.

## Conclusion

Sex influences in the response to treatment of patients suffering from HFrEF. All results from the landmark randomized clinical trials are based on study populations composed mainly of men. Further studies specifically designed to consider sex-differences are warranted to elucidate if GDMT and new devices are equally effective in both sexes.

## Data availability statement

The original contributions presented in the study are included in the article/supplementary materials, further inquiries can be directed to the corresponding author/s.

## Author contributions

MS and JÁ-G drafted the work. All authors made substantial contributions to the conception, design of the work, data acquisition, analysis, and interpretation of data for the work. All authors contributed to the article and approved the submitted version.

## Conflict of interest

The authors declare that the research was conducted in the absence of any commercial or financial relationships that could be construed as a potential conflict of interest.

## Publisher's note

All claims expressed in this article are solely those of the authors and do not necessarily represent those of their affiliated organizations, or those of the publisher, the editors and the reviewers. Any product that may be evaluated in this article, or claim that may be made by its manufacturer, is not guaranteed or endorsed by the publisher.
